# Multilayer Feature Extraction of AGCN on Surface Defect Detection of Steel Plates

**DOI:** 10.1155/2022/2549683

**Published:** 2022-10-03

**Authors:** Chi Zhang, Jian Cui, Wei Liu

**Affiliations:** ^1^Department of Industrial Engineering and Management, Peking University, Beijing 100871, China; ^2^China Mobile Research Institute, Beijing 100053, China

## Abstract

The development of industry is inseparable from the support of steel materials, and the modern industry has increasingly high requirements for the quality of steel plates. But the process of steel plate production produces many types of defects, such as roll marks, scratches, and scars. These defects will directly affect the quality and performance of the steel plate, so it is necessary to effectively detect them. Steel plate surface defects are characterized by their types, shape, and size: the same defect can have different morphologies, and similarities can exist between different defects. In this paper, industrial steel plate surface defect samples are analyzed, and a sample set is established by screening the collected defect images. Then, annotation and classification are performed. A multilayer feature extraction framework is developed in experiments to train a neural network on the sample set of defects. To address the problems of low automation, slow detection speed, and low accuracy of the traditional defect detection methods, the attention graph convolution network (AGCN) is investigated in this paper. Firstly, faster R-CNN is used as the basic network model for defect detection, and the visual features are jointly refined by combining attention mechanism and graph convolution neural network. The latter network enriches the contextual information in the visual features of steel plates and explores the semantic association between vision and defect types for different kinds of defects using the attention mechanism to achieve intelligent detection of defects, thus enabling our method to meet the practical needs of steel plate production.

## 1. Introduction

Steel is widely used in daily life and industrial production in a very large number of application areas and is the basic material for many products. According to statistics, in people's daily use of metal, steel accounts for up to 90% and is involved in most common products (home appliances, cell phones, etc.). The use of steel is essential in many industries, such as petrochemical, vehicle engineering, aerospace, military defense, and other fields, and its excellent performances have an invaluable role [1]. China's steel production in 2019 was as high as 996 million tons, accounting for 53.31% of the world's total production, far ahead of any other country [2].

Steel is an important and indispensable material in the modern construction of the country, and it is used in various production fields, especially in automotive, construction, and bridges. The production of steel plate is an extremely long and complicated process, from the raw stone to the final plate. It undergoes several processing steps, so the finished steel plate will inevitably have defects, most of them in the form of iron oxide, holes, cracks, scrapes, scratches, etc., on its surface. These defects directly affect the compressibility, toughness, corrosion resistance, and plasticity of the steel plates, rendering the manufactured products unable to meet customer requirements and resulting in severe economic losses for manufacturers. Without a set of effective testing methods, unqualified steel plate products put into use can even endanger people's lives and safety. Although steel production is high and export volumes are large, China still suffers from a slight shortage of automation compared to some developed countries [3]. Surface quality is a very important performance indicator of steel products, but it has not received the attention it deserves. According to statistics, because of surface quality problems, large steel industrial groups lose an average of about 6 million U.S. dollars a year because surface defects lead to the return of the products. According to the market response, the vast majority of companies are challenged by the presence of surface defects, causing huge economic losses and hindering the transformation and upgrading of enterprises. Hence, controlling steel surface defects at the source is a very effective measure. Therefore, steel plate manufacturers need to carry out effective quality inspection of their products, to screen the steel plates that do not meet the technical specifications and improve the yield rate of the delivered products. On the other hand, analyzing the causes of surface defects will provide a reliable basis for improving the steel plate production technology. However, it is difficult to detect surface defects on steel plates. Production is made in very harsh environments, so it is difficult to install and protect inspection equipment; furthermore, moisture and impurities increase the difficulty of inspection [4].

Many researchers have studied defect detection and proposed several effective methods, mostly based on manual visual inspection [5], magnetic particle detection, penetration detection, eddy current detection, ultrasonic detection, traditional machine vision detection and identification, and deep learning detection and identification methods. But the recent developments in artificial intelligence theory and technology [6], the emergence of high-speed, high-precision CCD and CMOS industrial cameras, and the tremendous increase of CPU and GPU computing power and distributed computing provide the theoretical basis and hardware conditions for high-speed high-precision detection of steel surface defects based on computer vision. Deep learning benefits from the recent progress in computing power and automation technologies. It is one of the most representative fields of artificial intelligence, with excellent performance in image classification, target detection, segmentation, and target tracking. Deep learning stands out in the field of related technologies with powerful memory capability, nonlinear mapping capability, self-learning capability, and robustness [7]. Training a deep learning network with a large amount of data enables the low-level network to automatically learn the detailed features in the data and the high-level network to automatically learn the abstract features. So far, deep learning techniques have been widely used in agriculture, medicine, automotive, and aviation. One of its important applications in industrial production is product quality estimation, where it efficiently overcomes the shortcomings of traditional defect detection methods, more sensitive to human and external environment interference. Deep learning-based methods can detect products defect more quickly and accurately.

The surface quality of a steel strip is an important indicator to evaluate the grade of steel. Surface defects not only affect its appearance, which is not conducive to sales and exports but also affect its mechanical properties and quality, decreasing its stiffness and strength and reducing its corrosion resistance. Defects may also be the cause of serious safety accidents. Analysis of the steel surface defects shows that there can be various kinds of defects on the steel surface, such as cracks, scratches, patches, inclusions, pitting, and bonds in the oxide skin, etc. In this paper, we propose a novel steel surface defect detection method (called AGCN) based on the attention mechanism and graph convolution neural network. The main contributions include the following: (1) the use of faster R-CNN [8] as the basic network model for surface defect detection and the combination of attention mechanism and graph convolution neural network; (2) exploring the contextual information in the visual features of steel plates and enhancing the semantic association between visual features and defect types using the attention mechanism and graph convolution neural network. Extended experiments are conducted on the steel surface dataset, and the advanced performance and effectiveness of the proposed method are demonstrated by method comparison and ablation analysis.

## 2. Related Work

### 2.1. Steel Plate Surface Defect Detection

The detection of steel surface defects began to develop in the 1920s and can be roughly divided into three families of methods: manual inspection, traditional photoelectric detection, and machine vision detection.

Manual inspection method, also known as manual visual method, was the first to be used. At that time, with the backward production technology, slow production speed, and low demand for products, the speed and quality of product testing were not very demanding. However, with the improvement in the level of production, the increase in demand and the disappearance of the demographic dividend, the shortcomings related to low inspection efficiency, high labor intensity, and nonuniform inspection standards gradually appeared and the inspection methods were no longer suitable for the requirements of speed and accuracy and were gradually abandoned [9].

Traditional photoelectric detection with optical sensors has been gradually applied in many enterprises, which improved the detection speed, accuracy, and efficiency [10]. Other methods based on eddy current detection and Faraday's electromagnetic induction principles have been developed. Common practice is to apply an alternating current to the strip surface, generating an alternating magnetic field affecting the detection coil. Measuring the induction current and impedance changes enables to determine the presence of defects. The main disadvantages are (1) waste of resources and (2) the method is not suitable for the detection of small defects. Leakage detection methods are based on the principle that when the steel plate is magnetized by a strong magnetic field, the change in the cross-sectional area of the steel plate due to the defect affects the magnetic permeability and reluctance. A part of the magnetic field bypasses the defective part through the surrounding air, causing the deformation of the magnetic field, from a straight line to a curve. The corresponding sensor converts the change of magnetic field into an electrical signal, and the size of the defect on the surface of the steel plate can be estimated from the electrical signal size. Leakage detection technology is a very simple, highly reliable, and fast detection technology, subject to small environmental interference factors. But at the same time, there are simple leakage signal characteristics, detection of defects, and a limited variety of disadvantages [11]. Infrared detection methods use the principle that any object continuously emits infrared waves depending on its temperature, according to Planck's law of radiation, Wien's displacement law, Stefan–Boltzmann's law, etc. Infrared detection is more functional and can be used for defect detection, stress and fatigue analysis, simulation, image processing and fault diagnosis, and some other functions. The detection device is made of three main parts: the excitation part (signal generator and excitation source), the infrared camera, and the PC terminal. The working process is roughly to apply the corresponding excitation source to the steel material to obtain its thermal phase diagram. Since the temperature of the defective and nondefective parts of the steel is not the same, this will form areas with different temperature levels on the steel surface and emit different infrared waves. However, this method can only detect a few defect types, so it cannot be widely used. Laser scanning detection uses a laser as the emitting light source. The laser beam is reflected on the surface of the strip towards a rotating reflector and finally through the optical system equipped with an electric multiplier tube which converts it into an electrical signal through the converter, so that it can ensure the detection of defects at a certain speed. Using upper and lower lasers, this method enables to scan simultaneously both sides of the strip and obtain two-sided data of the inspected material. The final image is then processed and analyzed by a computer. However, laser detection technology requires high environmental protection, allows only slow detection speed, continuous motion of the laser life and reliability is reduced, and the purchase and maintenance costs are high. The limitations are large, and it is difficult to make further breakthroughs. So many companies began to look for low cost, high detection efficiency, and easy maintenance detection equipment.

**Figure 1:**
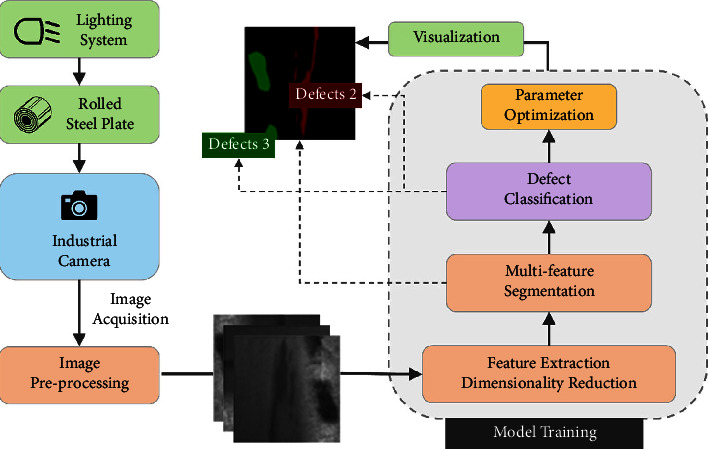
Process of computer vision diagnosis of surface defects on steel plates.

With the breakthrough of CCD (charge-coupled device) camera and related hardware, while computer technology gradually developed and gradually appeared in industries, online machine vision-based steel plate inspection became popular.  Figure 1 shows the working process and the role of each part: the steel plate to be inspected moves below the light source which provides additional light to remove the influence of background light and provide clear images collected by the camera which transmits them to the computer [12]. The computer analyzes and processes the images in real time according to the existing model and framework to assess the grade or defect situation of the steel plate surface. The use of CCD image sensors and pattern recognition technology has greatly improved the efficiency of steel surface defect detection, while various industries have started to detect surface defects with the help of similar devices.

Deep learning-based machine vision algorithms are the core of machine vision surface inspection technology, which is the key technology of the whole inspection system and one of the most challenging problems in the whole machine vision field. For steel surface defect detection, it is a popular research direction today to study algorithm models that can be executed with high accuracy, quickly extract the image features, and accurately identify the defect's category and location in real-time using massive image data. Deep learning is an end-to-end feature extracting algorithm, in which the model is similar to a black box. The process involves entering an image that contains a defect into the black box of the deep learning model which provides the category and location of the defect. Compared with traditional manual feature extraction, deep learning-based feature extraction enables more complete and accurate understanding of sample defects and features, thus achieving precise identification. Foreign research in this field began relatively early, for rail surface defect detection. The study and experiments of deep learning network models have concluded that different regularization methods have a certain impact on the recognition rate of defects. Sun et al. [13] designed a device for the identification of casting defects based on the mask R-CNN target detection model. Domestically, Cai and Wei [14] improved the YOLO target detection model with an accuracy of 97.55% in steel surface defect detection. He et al. [15] gave a multilayer feature fusion network structure using region proposal net (RPN) to generate regions of interest (RoI) on feature maps and obtained up to 82.3% of mAP (mean average precision) value on the dataset of defect detection at Tohoku University. To address the problem of insufficient dataset, Cui et al. [16] in foreign countries enhanced and expanded the dataset by cropping the original image, applying horizontal flipping, mirror flipping, transparency, and other processes. Liu et al. [17] used GAN (generative adversarial networks) network models to generate new defective dataset sample by merging original defect samples and defect-free samples and expand the dataset and achieve the purpose of sample migration. From the above analysis, with the increase of steel production, the defect detection of steel surface has stepped into the era of intelligent detection.

### 2.2. Multilayer Feature Extraction

Convolutional neural networks (CNN) have developed rapidly and caught everyone's attention with their powerful modeling capabilities. Compared with traditional methods, the introduction of CNN has brought great improvements to areas such as image processing and natural language processing, for automatic translation, image, and speech recognition. However, traditional CNNs can only process Euclidean space data (e.g., images, text, and speech), which are translation invariant in these domains [18]. Translational invariance allows us to define convolution networks by defining globally shared convolution kernels in the input data space. Taking image data as an example, a picture can be represented as a set of regularly spaced pixels in the Euclidean space, and translation invariance means that a local structure of the same size can be obtained around any pixel [19]. Based on this, CNNs model local connectivity by learning convolution kernels shared at each pixel, which in turn create meaningful hidden layer representations for pictures. Although traditional CNNs bring enhancements in text and image domains, they can only handle Euclidean space data. Meanwhile, non-Euclidean spatial data—graph data—are gradually gaining attention due to its ubiquity [20].

**Figure 2:**
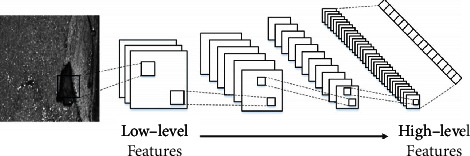
Feature extraction from low level to high level.

In defect segmentation, the model needs to extract sufficient and effective semantic information to describe the difference between foreground (refer to the defects) and background (refer to the noise). Mahendran and Vedaldi [21] considered that convolution network feature maps in different layers contain the characteristics of different context information abundance. As shown in Figure 2, low-level feature maps with high resolution have clearer edges more detail information, which can be used to describe specific texture feature, but it contains less context information. On the contrary, the context information of high-level features is more abstract, and the semantic information is more separable after multilayer convolution extraction, but the texture details cannot be extracted due to low resolution. For classification problems, most methods mainly focus on high-level features, resulting in poor defect segmentation results in complex backgrounds. Inspired by the multilayer feature fusion method [22], this paper introduces the boundary refinement module to retain the low-level texture information.

Besides, the segmentation models need to be nondeformable for various variations such as defect's shape, scale, and texture. Most CNN-based methods try to expand the receptive field to cover the entire defect for global perception. In the DeepLab model [23], the receptive field is extended in the last convolutional layer to enhance the recognition of feature changes, but this will lead to grid artifacts [24]. Zhao et al. [25] use pyramid models with different pooling cores to amplify local features to overcome intraclass differences. However, excessive pooling in feature fusion makes the model unable to capture a wider range of global information, resulting in missing parts when marking defect masks [26]. To solve the above problems, we propose a multilayer feature fusion method, which uses multiscale convolution (receptive fields of different sizes) to weight the feature maps of all convolutional layers to obtain the context information. On the premise of fully exploiting defect features, grid artifacts and excessive pooling are avoided.

### 2.3. Graph Convolution Neural Network

Graph data can naturally represent real-life data structures, such as traffic networks, World Wide Web, and social networks. Unlike image and text data, the local structure of each node in graph data varies, which makes translation invariance no longer satisfied. The lack of translation invariance poses a challenge to define CNN on graph data. In recent years, due to the prevalence of graph data, researchers have started to focus on how to construct deep learning models on graphs. With the ability of CNNs to model local structures and the prevalence of node dependencies on graphs, GCN (graph convolution neural) networks have become one of the most active and important research fields. Recently, several articles have been published to explore deep learning on graphs, but there is still a gap in the in-depth discussion and summary of the modeling methods and applications of the most important branch, graph CNNs. In this paper, we summarize the development of GCNs and their future trends [27].

The challenges faced in the construction of GCNs are mainly related to the following aspects: (1) graph data are non-Euclidean spatial data and do not satisfy translation invariance, i.e., each node has a different local structure. The basic operators in traditional CNNs (convolution and pooling) rely on the translation invariance of the data. At this point, it becomes a challenging task to define convolution and pooling operators on graph data. (2) A variety of real-life applications can be naturally represented by graphs, which give them diverse properties, such as directed connections of users in social networks, heterogeneous connections of authors and citations in citation networks, and positive and negative tendency band symbolic connections in political relationship networks. The various graph characteristics bring more information to the construction of GCNs, but the modeling of multiple characteristics also requires a more complex and detailed design of GCNs, which brings new challenges. (3) The scale of graph data is very large: in the era of big data, graphs in practical applications may be extremely large, with millions or even tens of millions of nodes, such as user commodity networks in recommendation systems and user networks in social networks. It is very challenging to build GCNs on large-scale graphs in an acceptable range of time and space [28].

In addition, researchers borrowed knowledge from graph theory, such as using eigenvalues and eigenvectors of Laplacian matrices for community analysis or population clustering. With the rise of deep learning, researchers started to consider introducing deep learning models into graph data, and the representative research work is called network embedding, i.e., learning fixed-length expressions for each node by constraining the proximity of nodes. This led to new methods such as Deep Walk, LINE, and node2vec. During this period, when solving specific application problems, researchers usually modeled them as two-stage problems [29]: taking node classification as an example, the first stage learns uniform-length expressions for each node, whereas the second stage uses node expressions as inputs to train classification models. In recent years, researchers have gradually shifted their focus from modeling graph data to how to migrate deep learning models to graphs for end-to-end modeling, and GCNs are one of the most active fields. In modeling graph convolution neural networks, researchers focus on how to build convolution operators on graphs. Zhang et al. [30] proposed the first graph convolution neural network in 2013, where they defined graph convolution in the spectral space based on graph theory using the convolution theorem. This branch was later developed as the spectral approach in the field of graph convolution. The initial spectral methods had the disadvantage of high spatio-temporal complexity, and Cheb-Net and GCN parametrized the convolution kernel in the spectral domain to greatly reduce the spatio-temporal complexity [31]. These two methods, although categorized as spectral methods, have started to define the weight matrix of nodes from a spatial perspective. Inspired by these two methods, spatial methods were applied and began to consider modeling the weights between nodes in the node domain with attention mechanisms, serialization models, etc. The graph convolution neural networks of this period did not take too much account of the characteristics of graphs in the process of constructing convolution operators. With the gradual improvement of convolution operators, people began to consider various features of graphs, starting with a focus on how to model higher-order information on graphs, and fine-grained designs for graphs with features on edges, heterogeneous graphs, etc. In addition, the question of how to train more efficient GCNs has also received much attention. Researchers have started to train deeper GCNs to enhance generalization. In addition, the scalability of the models to large-scale graphs and the training speed are very focused research directions in GCN. The pooling operator, as the main component of CNNs, enables to expand the perceptual field and reduce the number of parameters. Recently, some research has also started to focus on the construction of on-graph pooling operators [32]. The on-graph pooling operator is mainly used in graph classification problems with the aim of learning the hierarchical structure of the graph. The broad application scenarios targeted by graph data modeling makes the tasks handled by graph data modeling diverse. We divide the downstream tasks into node-level tasks and graph-level tasks. Node-level tasks include node classification and link prediction, such as article classification in citation networks and inference of user preferences for products in recommendation systems. Graph-level tasks include graph generation and graph classification, such as drug network generation and protein classification in protein networks [33].

## 3. Method

### 3.1. Steel Plate Defect Analysis

**Figure 3:**
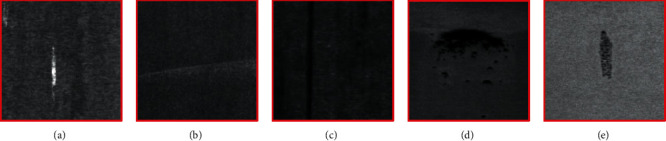
Types of defects in the collected data set. (a) White iron scale. (b) Roll printing. (c) Scratch. (d) Scarring. (e) Embroidery skin.

Since the original size of each defect can vary greatly, we will apply scaling to achieve a uniform size.  Figure 3 shows five common steel plate surface defects: their basic characteristics are as follows. (1) White iron scale: mainly in the form of strips of varying length, the color is generally white, mostly in patches of aggregated distribution, and the size of the defect varies. (2) Roll marks include three main types of features: defects for the lighter gray-white distribution of scattered microarcs, with low contrast to the background; defects for the dark gray arc-shaped microfolds; and a small number of defects for the continuous gray-black periodic straight band. (3) Scratches are generally gray-black, mainly in the width of the continuous periodic band. When the background color is dark, the defect looks like it and the contrast is low. (4) Scarring: mainly in the form of black dots or surfaces of different sizes, usually aggregated in patches, part of the distribution is more scattered. The background brightness may vary: as the background gets darker, the contrast of features gradually decreases. (5) Rusty skin/embroidery skin. Mainly a certain width of short gray-black bands, features are more obvious, usually a single distribution or a very small number of clustered distributions. The above five kinds of defects are the ones that are the most studied.

The analysis of a large number of defect images shows that there are similarities between different types of defects (such as interrupted parts of rusty skin and scars, shallower, smaller scars, and white iron scales), and the same type of defects have a variety of forms and sizes (roll printing defects have three different forms and sizes). Traditional vision inspection methods have difficulty solving these problems, while GNN-based inspection algorithms can effectively detect these complex forms of defects.

### 3.2. Model Architecture

**Figure 4:**
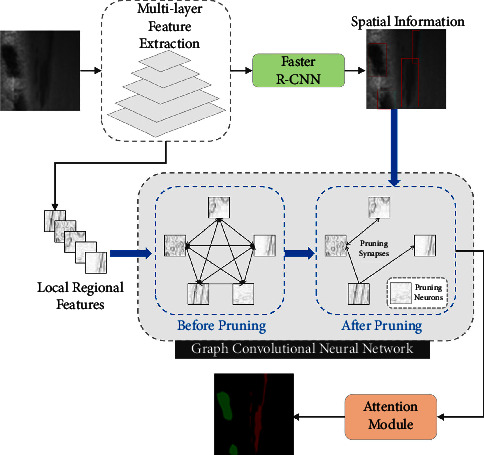
Model architecture.

The proposed model is shown in Figure 4 and is made of three parts: (1) multilayer feature extraction network as backbone for steel plate defect detection, to extract visual features and spatial information of salient regions. (2) Graph CNN: to enrich the contextual information of visual features. (3) Attention mechanism: to explore the semantic association between visual features and fault categories.

### 3.3. Multilayer Feature Extraction

The multilayer feature extraction module is divided into 4 parts, which is feature extraction network, RPN (region proposal network) module, RoI pooling module, and R-CNN module [34]. It mainly generates candidate regions and performs preliminary classification and localization through RPN [35]. Then, it pools the acquired candidate regions and finally classifies and again improves the positions of the pooled defect features.

**Figure 5:**
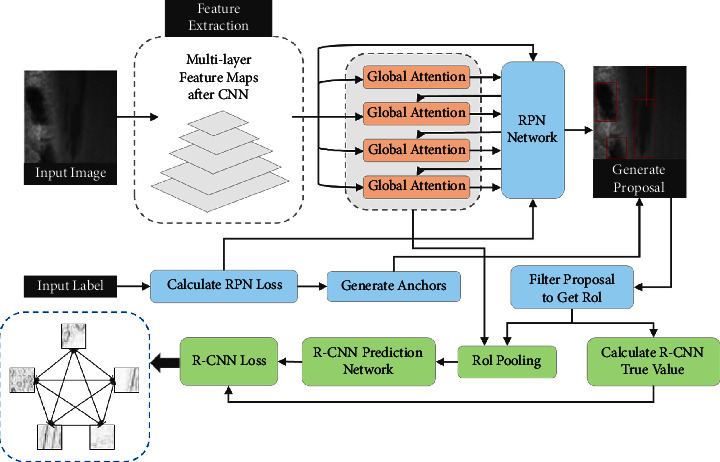
Schematic diagram of the multilayer feature extraction process.

In this paper, firstly, we establish the global context attention mechanism into the adjacent resolution feature map. Secondly, the global context information is extracted from the low-resolution feature map. Thirdly, the high-resolution information is weighted to refine the spatial position of the category pixels, which can ensure the high-level features are not weakened and achieve a more accurate classification result without increasing the amount of calculation. Consider RPN network can map the generated region to the feature map generated by the convolution network through “anchors,” realizing the connection between the two and further improving the detection speed and accuracy [36]. To learn whether a defect is present in the input image, anchors (rectangular boxes with a certain size and aspect ratio) are placed on the image for each location on the output feature map from the RPN network. Then, the anchor is matched with the real defect, and the classification and fine-tuning of the defect location is performed.  Figure 5 shows the computational flowchart of the multilayer extraction process, dividing the detection process in two steps and providing preliminary localization and classification (proposal). The more accurate the proposal is, the smaller the error of the later redetection. The RoI is obtained by screening a large number of proposals generated by the predicted anchor during training, and the proposal is directly used as the RoI during testing. RPN module mainly consists of five submodules:

1. Anchor generation: RPN corresponds to nine anchors for each point on the generated feature map, and each anchor has three different area sizes and three different aspect ratios, corresponding to the original map covering possible defects.
2. RPN convolution network: by employing a convolution network, each generated anchor is processed to obtain its prediction score and offset value.
3. Calculate RPN loss: this part occurs only during the training process, matching the anchor with the labels to distinguish between positive and negative samples, obtaining the true values of classification and offset, and calculating the loss with the prediction score and offset values obtained in the previous step.
4. Generate proposal: screen the anchor obtained by the RPN convolution network to get a better set of proposal for the subsequent network.
5. Screening proposal to get RoI: screen the proposal obtained in the previous step to get the final RoI.

#### 3.3.1. Feature Extraction Network

In order to obtain a better feature map if the image has a low contrast, a 13-layer convolution network similar to VGG16 is used to extract the defect features of the scaled image. The convolution layers use a small convolution kernel of size 3 × 3 with a small number of parameters, while the large number of layers gives it a better nonlinear capability to improve its learning capacity. This part includes five convolution modules, each outputting feature maps of 64, 128, 256, 512, and 512, respectively.

#### 3.3.2. RPN Module

Anchor generation is performed on the feature maps, the category score, and position offset value of each anchor are predicted, and the binary classification of defects (i.e., the presence or absence) and their preliminary location are performed according to the acquired proposals [37]. This module shares the convolution features of the whole map with the R-CNN detection network, saving time and providing high quality proposals to the R-CNN detection network, which improves the detection accuracy of the model. The number of output feature maps is 512, and 18 and 36 in the later classification and localization parts, respectively.

#### 3.3.3. RoI Pooling Module

Since the R-CNN module behind the faster R-CNN uses a fully connected layer, a uniform dimension is required before the defect feature subgraphs are input to the fully connected layer (the feature subgraph size in this paper is 7 × 7). Since the defect images of steel plates are of different sizes, their RoI corresponding feature map sizes are also different. RoI pooling is used to perform feature scale transformation to enable taking defect images of arbitrary sizes as input and output a fixed feature map size, suitable for detecting the five different defects scales of this paper. The number of feature maps in this part is kept constant.

#### 3.3.4. R-CNN Module

The RoI feature subgraphs obtained from RoI pooling are mapped to the whole feature map and output to the fully connected network of the R-CNN module, which performs the defect class prediction and location regression. This part of the fully connected layer outputs 2048 feature maps. Faster R-CNN outputs the visual features of the *i*-th anchor point as *h*={*h*_*i*_}_*i*=0_^*m*^ ∈ *R*^*m*×2048^ and the spatial information as *S*_*i*_=[*x*_*i*1_, *y*_*i*1_, *x*_*i*2_, *y*_*i*2_], where *m* denotes the number of salient regions.

### 3.4. Graph Convolution Neural Network

**Figure 6:**
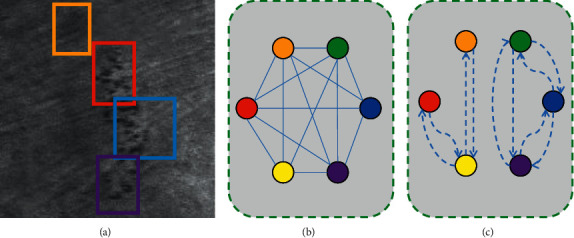
Graph generation.

A first fully connected graph is constructed and further refined using the accurate contextual information between multiple salient regions, to obtain a spatial graph network. We use faster R-CNN to obtain the visual features and spatial information of saliency regions, as shown in Figure 6(a). Next, we introduce the construction of the fully connected graph by considering each object region in the image as a vertex, and by constructing a relationship graph, we obtain a fully connected indirect graph as shown in Figure 6(b), where each edge represents the relationship between two regions. The spatial information of the regions representing the position of the regions in the image is a four-dimensional spatial vector*S*_*i*_=[*x*_*i*1_, *y*_*i*1_, *x*_*i*2_, *y*_*i*2_], where (*x*_*i*1_, *y*_*i*1_) is the coordinate of the upper left corner of the bounding box and (*x*_*i*2_, *y*_*i*2_) is the coordinate of the lower right corner of the bounding box. The identification of the correlations between regions is done according to the following steps: (1) the visual features of the two regions are fed into the multilayer perceptron to obtain feature integration, and the corresponding elements of the two-feature embedding are multiplied to obtain a correlation score. (2) We determine whether there is a correlation between two regions based on the size of the overlap area. If two regions have a large overlapping area, it means there is a strong correlation between these two regions. If the two regions do not have any overlapping part, we consider that these two regions have weak correlation, which means there is no edge connecting these two nodes. In addition, we identified five different categories of region relationships, such as internal, overlay, and overlap. Based on the spatial relations, we removed some irrelevant region relations from the fully connected graph and obtained a relatively sparse graph as shown in Figure 6(c).

To enhance the contextual information in the visual features of each region, we use GCN to update the object representation. If the image contains *m* salient regions, considered as nodes, we use the *m* × *m* adjacency matrix *A* to represent the structure of the graph, with *A*_*ij*_=1 if there is an overlapping region between node *i* and node *j*; *A*_*ij*_=0 otherwise. Given the target node *i* and neighboring node *j* ∈ *N*(*i*) in the image, where *N*(*i*) is the set of nodes adjacent to node *i*, the visual feature representations of node *i* and node *j* are *h*_*i*_ and *h*_*j*_, respectively. To obtain the correlation score *s*_*ij*_ between nodes *i* and*j*, by splicing the visual features of *h*_*i*_ and *h*_*j*_, we first train a fully connected layer.

(1) $$\begin{array}{c} s_{ij}=w_{a}^{T}\sigma \left(W_{a}\left[h_{i},h_{j}\right]\right)\text{,} \end{array}$$

where *w*_*a*_ and *W*_*a*_ are the learning parameters, *σ* is the nonlinear activation function, and [*h*_*i*_, *h*_*j*_] denotes the concatenation operation. We apply the softmax function on the correlation score *s*_*ij*_ to obtain the weight *α*_*ij*_, as shown in Figure 6(c).

(2) $$\begin{array}{c} \alpha_{ij}=\frac{\exp\;\left(sij\right)}{\sum_{j\in N\left(i\right)}\exp\;\left(sij\right)}\text{.} \end{array}$$

For the graph convolution, the neighboring nodes of *h*_*j*_, *j* ∈ *N*(*i*) are first passed through a learned linear transformation *W*_*b*_. These transformed representations are aggregated by the weights *α*_*ij*_, and finally the updated node features *h*_*vi*_ are obtained by the activation function *σ*:

(3) $$\begin{array}{c} h_{vi}=\sigma \left(h_{i}+\underset{j\in N\left(i\right)}{\sum }A_{ij}\alpha i_{j}W_{b}h_{j}\right)\text{.} \end{array}$$

The output feature of the last layer node *i* in GCN is *H*_*i*_, and the set of features of all nodes is *H*.

### 3.5. Attention Mechanism

In order to enhance the higher-order semantic association between visual features and defect types, to refine and reduce the redundant information in visual features and highlight the key semantics, we designed the attention mechanism. First, for the regional visual representation *H* obtained by convolution of the graph neural network, each node's feature set is updated using the self-attention mechanism to obtain a new feature set $\tilde{H}$:

(4) $$\begin{array}{c} \tilde{H}=softmax\left(\frac{{HH}^{T}}{\sqrt{d}}\right)H\text{,} \end{array}$$

where *H*^*T*^ is the transpose of *H* and *d* is the dimension of *H*. To obtain the visual representation associated with the surface defect representation of the steel plate, we utilize the design learnable parameter matrices *W*_*m*_. *W*_*m*_ is used as a guiding matrix to adjust the visual representation $\tilde{H}$. The similarity score between *W*_*m*_ and $\tilde{H}$ is calculated as follows:

(5) $$\begin{array}{c} r=\frac{W_{m}{\tilde{H}}^{T}}{\sqrt{d_{m}}}\text{.} \end{array}$$

For the *i*-th region, a softmax function is used to normalize the score *ri* to obtain the defect diagnosis probability ${\tilde{r}}_{i}$.

(6) $$\begin{array}{c} \tilde{r}=\left[{\tilde{r}}_{1},{\tilde{r}}_{2}\ldots ,{\tilde{r}}_{i}\right]=\frac{\exp\left(r_{i}\right)}{\sum_{j\in m}\exp\left(r_{j}\right)}\text{.} \end{array}$$

### 3.6. Loss Function

We deploy a multitask loss function that combines classification loss and edge localization regression loss for unified training and finally outputs the corresponding classification and edge locations, which can improve the detection accuracy and is suitable for detecting small target defects:

(7) $$\begin{array}{c} L\left(\left\{{\tilde{r}}_{i}\right\},\left\{S_{i}\right\}\right)=\frac{1}{N_{cls}}\underset{i}{\sum }L_{cls}\left({\tilde{r}}_{i},r_{i}^{*}\right)+\lambda \frac{1}{N_{reg}}\underset{i}{\sum }p_{i}^{*}L_{reg}\left(S_{i},S_{i}^{*}\right)\text{,} \end{array}$$

where *i* is the index of the anchor; ${\tilde{r}}_{i}$ is the probability of anchor being predicted as a target; *r*_*i*_^*∗*^ is the category label, 1 when a target exists and 0 otherwise; *S*_*i*_ is the predicted value of location regression; *S*_*i*_^*∗*^ is the label value of location regression; *λ* is the weight balance value; *N*_cls_ and *N*_reg_ are the normalized values of classification loss *L*_cls_ and regression loss *L*_reg_, respectively.

## 4. Experimentation and Evaluation

### 4.1. Datasets

We set up a test bench in a steel production factory and used a line-scan camera to acquire online images of steel plates and obtained a total of 5,000 defect samples after screening, 1,000 for each defect type, of which 4,000 were used as training samples and 1,000 were used as test samples. In the datasets of this paper, each defect image contains at least one defect, and some images contain multiple defects of different scales to ensure that the trained detection model can adapt to complex detection [38]. In this paper, the defect samples are labeled with defects using labeling software with rectangular boxes, where the label name is the first letter of the Chinese pinyin capitalization of each defect, such as TL for white iron scale, GY for roll marks, GH for scratches, JB for scars, and XP for rusty/embroidery skin.

### 4.2. Experimental Setup and Evaluation Metrics

**Figure 7:**
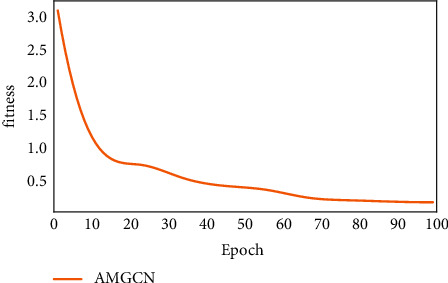
Training loss convergence plot.

The experiments were completed using the conditions shown in Table 1. To further demonstrate the effectiveness of the proposed method, 6 models and the AGCN were trained for 100 epochs (rounds) on the datasets of this paper. The training methods are as follows: the initial learning rate of the model is set to 0.0001; the Adam optimizer is used; the learning rate decay is performed once every 5 epochs, and its decay rate is 0.1; the batch size is set to 8 (the batch size is the number of samples selected for one training epoch; it is limited by the GPU of the device, and chosen to obtain the best optimization and highest training speed). The loss value plot of the proposed model trained on the dataset of this paper with 100 epochs is shown in Figure 7, where the vertical coordinate is the loss value (loss) and the horizontal coordinate is the number of training sessions. The loss values converge quickly during the training process and finally sets to about 0.18.

### 4.3. Performance Comparison

In this paper, the average accuracy mIoU (mean intersection over union) and frame rate are used as the actual metrics for defect detection, and the final saved optimal network model is taken for testing. The average detection accuracies of the five defects on the test set are shown in Table 2.

In order to avoid overfitting, this paper adopts 5-fold cross-validation. Compared with the original method (faster R-CNN) and recent advanced methods, our method has the highest average mIoU of 0.8580. Among the existing five categories of defect recognition, our methods are the most prominent in the 4 categories of defects. Compared with the most accurate RefineNet model, we are only 0.0092 behind in the defect detection of roll printing. It is proved that this method is effective for steel plate defect detection.

On the other hand, the recognition accuracy of each category of defects is different. Since embroidery skin is generally reflective which have large contrast with the background noise, its features are easy to be captured by the algorithm, and its recognition accuracy is generally higher than that of other categories. For roll printing, it typically appears as a long strip. However, the length of such defects varies in different images. One picture probably has more than one roll printing defects, which leads to low mIoU.

From Tables 2 and 3, it can be seen that faster R-CNN obtains a better detection accuracy than YOLOv4 on the test set; the defect detection accuracy of AGCN is better than that of faster R-CNN. AGCN improves the average detection accuracy of embroidery skin defects to 97.67%, which is 7.5% better compared with faster R-CNN. Compared with faster R-CNN and YOLOv4 model, the detection time of AGCN model is increased, but the impact is not significant, and our model can meet the practical requirements. The trained model is tested on the test set and found to have issues such as false detection and missed detection. Defect characteristics vary greatly between classes, and when several types of defects have similarities, the accuracy is low. If a single “White iron scale” defect is present in a small area of an image, after multiple convolutions, the model losses the defect characteristics and cannot detect it. For “Roll marks” defects, when the color is light, bright or white, this type of defect can be misdiagnosed as “White iron scale”; because when the color is very similar to the background, the characteristics are not obvious and detection can be missed. When several small target defects are detected in an image, such as rusty/embroidery skin defects, the AGCN model has significantly higher average detection accuracy (The segmentation map are shown inFigure8).

### 4.4. Ablation Experiments

To verify the role of the proposed attention module and the graph convolutional neural network, we did some ablation experiments. The number of convolutional layers of the graph convolutional neural network is varied to verify the effect of the model. We set the number of convolution layers as 1, 2, 3, and 4. The results are shown in Table 4. The model achieves the best results when the number of convolution layers is 3, which indicates that there are deep semantic associations between different salient regions in the images that require multilevel graph convolution. The effectiveness of the proposed graph convolutional neural network is demonstrated.

## 5. Conclusion

This paper introduces a AGCN detection method based on the multilayer feature. We analyze the features of the most common steel plate defects and the characteristics of the faster R-CNN network-based model as the visual multifeature encoder and propose to refine the visual features by combining the attention mechanism and the graph convolution neural network approaches. The graph convolution neural network enriches the contextual information in the visual features of steel plates and further explores the semantic association between vision and defect types using the attention mechanism to achieve intelligent defect detection. This method meets the practical needs of defect detection in steel plate production. In the future, we plan to explore the dynamic neural network-based steel plate defect detection.

## Figures and Tables

**Figure 8:**
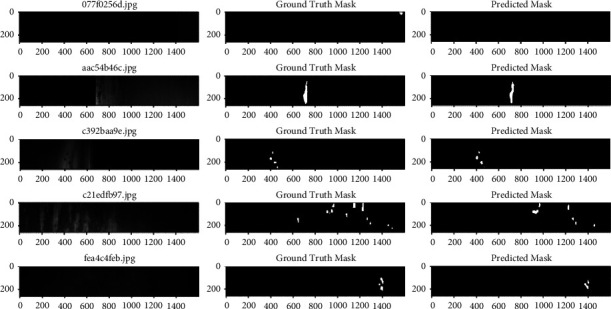
Partial experimental results—segmentation map.

**Table 1:** Experiments conditions.

CPU	Intel Xeon W-2135
Operating system	Ubuntu 18.04
RAM	32 GB
GPU	GeForce RTX 2080Ti
Video memory	8 GB
Python version	3.6.9
CUDA	10.0
CU DNN	7.4.1

**Table 2:** Performance comparison (mIoU).

Model	White iron scale	Roll printing	Scratch	Scarring	Embroidery skin	Average
Faster R-CNN [8]	0.7962	0.7204	0.8386	0.7602	0.8982	0.8027
SegNet [39]	0.8250	0.6835	0.8532	0.8622	0.8824	0.8213
PSPNet [40]	0.8032	0.7282	0.8419	0.8358	0.9047	0.8228
YOLOv4 [14]	0.8068	0.7025	0.8793	0.8524	0.8856	0.8253
DeepLab+ [23]	0.8271	0.7139	0.8748	0.8437	0.8754	0.8270
RefineNet [41]	0.8265	**0.7280**	0.8786	0.8613	0.8410	0.8271
AGCN (ours)	**0.8485**	0.7188	**0.9098**	**0.8762**	**0.9367**	0.8580

**Table 3:** Average detection speed.

Model	Testing time in seconds	Number of frames per second
Faster R-CNN	0.0621	16.11
SegNet	0.0464	21.54
PSPNet	0.0427	23.42
YOLOv4	0.0340	**29.39**
DeepLab+	0.0386	25.92
RefineNet	0.0534	18.73
AGCN (ours)	0.0364	**27.46**

**Table 4:** Ablation experiments.

Number of convolutional layers	White iron scale (%)	Roll printing (%)	Scratch (%)	Scarring (%)	Embroidery skin (%)
1	82.72	72.11	90.87	89.08	90.89
2	82.75	72.24	90.86	89.32	93.82
3	**84.85**	**72.88**	**90.98**	**89.62**	**97.67**
4	83.62	72.54	90.76	89.42	95.82

## Data Availability

The datasets used during the current study are available from the corresponding author on reasonable request.
